# LncRNA SRA promotes hepatic steatosis through repressing the expression of adipose triglyceride lipase (ATGL)

**DOI:** 10.1038/srep35531

**Published:** 2016-10-19

**Authors:** Gang Chen, Dongsheng Yu, Xue Nian, Junyi Liu, Ronald J. Koenig, Bin Xu, Liang Sheng

**Affiliations:** 1 Department of Hepatobiliary Surgery, The First Affiliated Hospital, Wenzhou Medical University, Wenzhou 325000, China; 2 Department of Pharmacology, School of Basic Medical Science, Nanjing Medical University, 140 Hanzhong Rd., Nanjing, Jiangsu, 210029, China; 3 Department of Chemical Biology, School of Pharmaceutical Sciences, Peking University, Beijing 100191, China; 4 Department of Internal Medicine, Division of Metabolism, Endocrinology and Diabetes, University of Michigan Medical Center, Ann Arbor, MI 48109-5678, USA.

## Abstract

Nonalcoholic fatty liver disease (NAFLD), the most common form of chronic liver disease, manifests as an over-accumulation of hepatic fat. We have recently shown that mice with genetic knockout of a long non-coding RNA (lncRNA) steroid receptor RNA activator (SRA) (SRAKO) are resistant to high fat diet-induced obesity with a phenotype that includes improved glucose tolerance and attenuated hepatic steatosis. The underlying mechanism was investigated in the present study. We found that hepatic levels of SRA and adipose triglyceride lipase (ATGL), a major hepatic triacylglycerol (TAG) hydrolase, were inversely regulated by fasting in mice, and the expression of liver ATGL was induced by SRAKO under normal and high fat diet (HFD) feeding. Loss of SRA in primary hepatocytes or a hepatocyte cell line upregulates, but forced expression of SRA inhibits ATGL expression and free fatty acids (FFA) β-oxidation. SRA inhibits ATGL promoter activity, primarily by inhibiting the otherwise-inductive effects of the transcription factor, forkhead box protein O1 (FoxO1). Our data reveal a novel function of SRA in promoting hepatic steatosis through repression of ATGL expression.

Nonalcoholic fatty liver disease (NAFLD) occurs when fat is over-deposited in the liver in the absence of excess alcohol intake, and is associated with obesity, insulin resistance, and metabolic syndrome^1^,^2^. NAFLD is usually caused by an imbalance between hepatic synthesis and breakdown of fats, as well as free fatty acids (FFA) storage and disposal. Nonalcoholic steatohepatitis (NASH), the more virulent form of NAFLD, can lead to cirrhosis. NAFLD is the most common liver disorder in developed countries^3^,^4^. A recent study using the National Health and Nutrition Examination Survey found a 30% prevalence of NAFLD in the United States between 2011 and 2012^5^. Normal hepatic lipid metabolism relies on the functional coordination of multiple physiological processes, including lipogenesis, FFA β-oxidation, lipid uptake, very low density lipoprotein (VLDL) secretion and lipolysis. These processes are tightly controlled by genes such as *Srebp1c* (lipogenesis), *peroxisome proliferator-activated receptor* *α* (*Pparα*) (FFA β-oxidation), *Cd36* (lipid uptake), *apolipoprotein B* (*apoB*), *microsomal triglyceride transfer protein* (*MTTP*) (VLDL secretion), and *Pnpla2* (encoding adipose triglyceride lipase, ATGL) (lipolysis)^6^,^7^,^8^. The functions of these genes are further modulated by different biological incidents, including ligand binding, transcription, posttranscriptional modification and protein degradation, which are controlled by metabolites and hormones. Among those genes, ATGL is particularly important since ATGL is a major hepatic triacylglycerol (TAG) hydrolase^8^,^9^. Interestingly, ATGL knockout mice exhibit increased glucose tolerance and insulin sensitivity with enhanced insulin signaling in skeletal muscle and white adipose tissue, but attenuated insulin signaling in liver and brown adipose tissue^10^,^11^.

Recently, long non-coding RNAs (lncRNAs) have emerged as important regulators of diverse biological processes including stem cell pluripotency, embryogenesis, cellular differentiation^12^,^13^, and hepatic lipid metabolism^14^. Steroid receptor RNA activator (SRA) was initially characterized as a lncRNA that functions as an RNA coactivator to enhance steroid nuclear receptor-dependent gene expression^15^. Subsequently, SRA also was demonstrated to function as an RNA coactivator for non-steroid nuclear receptors^16^,^17^, and to play important roles in myogenesis^18^,^19^, steroidogenesis^20^, breast tumorigenesis^21^,^22^,^23^ and cardiomyopathy^24^. The *Sra1* gene also produces an alternative transcript that encodes a protein denoted as SRAP^25^,^26^, although the function of SRAP is largely unknown.

We have recently shown that SRA promotes adipocyte differentiation and insulin-stimulated glucose uptake in adipocytes *in vitro* through multiple mechanisms, such as coactivating the transcriptional activity of peroxisome proliferator-activated receptor γ (PPARγ), inhibiting expression of adipocyte-related inflammatory genes or promoting insulin receptor expression^27^,^28^. To assess SRA function *in vivo*, we generated global *Sra1* gene knockout mice (SRAKO)^29^. In addition to reduced fat mass, SRAKO mice have decreased hepatic TAG levels and resistance to diet-induced obesity. These data, for the first time, indicate a role for SRA in hepatic lipid metabolism^29^. Here we elucidate the mechanism by showing that SRA inhibits the transcriptional activity of forkhead box protein O1 (FoxO1) via an insulin-independent pathway in hepatocytes, thus reducing the expression of its downstream gene ATGL, which is a key lipolytic enzyme, and subsequently decreasing hepatocyte FFA β-oxidation.

## Results

### SRA deficiency upregulates the hepatic expression of ATGL

**Figure 1:**
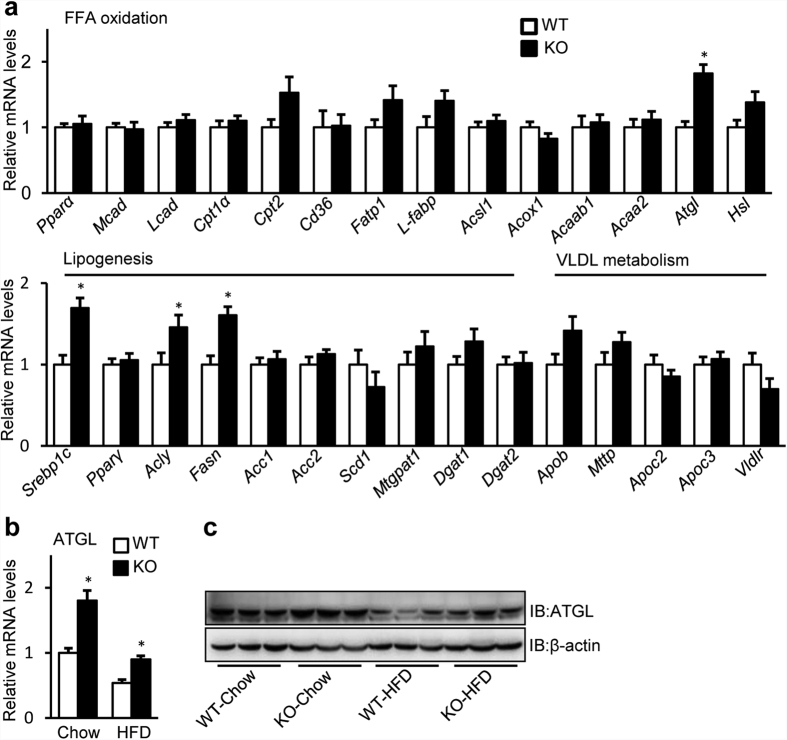
Loss of SRA increases the hepatic ATGL expression. (**a**) RT-qPCR analysis of mRNA expression of FFA oxidation, lipogenesis and VLDL metabolism-related genes in livers of male SRAKO (KO, n = 8) or WT (n = 8) littermates (20 weeks of age) fed with normal chow. (**b**) Hepatic mRNA level of ATGL in SRAKO (KO, n = 8) or WT (n = 8) littermates (20 weeks of age) fed with normal chow or HFD. The mRNA levels of genes were normalized to 36B4 expression. The data are presented as the mean ± SE and expressed as fold-change relative to the level of WT-Chow. *p < 0.05. (**c**) Liver lysates from male SRAKO (KO) or WT littermates (20 weeks of age) under different diet conditions were immunoblotted with anti-ATGL and anti-β-actin antibodies.

We have previously shown that SRAKO mice are resistant to diet-induced obesity with reduced fat mass, and have a phenotype of mild lipodystrophy^29^. Unlike the usual lipodystrophy with hepatic steatosis, insulin resistance and inflammation due to poor storage of TAGs in adipocytes^30^, SRAKO mice have reduced inflammation, improved insulin sensitivity and decreased hepatic TAG contents^29^. One potential explanation is that SRAKO may prevent hepatic steatosis by increasing FFA β-oxidation in liver^31^. To identify key regulator(s) that may lower hepatic TAG content, we examined the expression of hepatic genes that regulate FFA β-oxidation, lipogenesis and VLDL secretion in SRAKO mice fed with normal chow. As shown in Fig. 1a, liver mRNA levels of most of the genes responsible for FFA β-oxidation were unchanged, but the expression of ATGL was upregulated. No downregulation of genes responsible for lipogenesis or VLDL metabolism was seen, and in fact, Srebp1c, Acly and Fasn were upregulated by SRA deficiency. However, we found that the mRNA levels of Srebp1c, Acly and Fasn were unchanged in cultured primary hepatocytes of SRAKO mice and were uninfluenced by overexpression of SRA or SRAP in the mouse hepatocyte cell line, Hepa1–6 (Supplementary Figure S1), suggesting that the changes in Srebp1c, Acly and Fasn expression seen in Fig. 1a are likely secondary effects of the *in vivo* situation. This contrasts with the direct regulation of ATGL by SRA described below.

**Figure 2:**
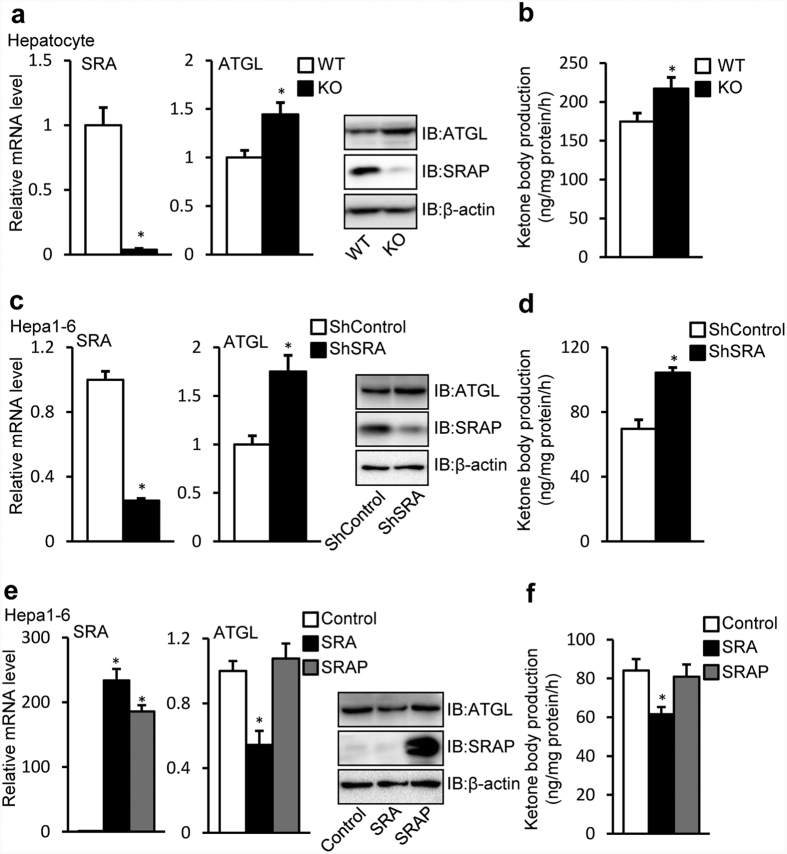
SRA inhibits FFA oxidation by repressing ATGL expression in hepatocytes. (**a**) SRA and ATGL mRNA levels in primary hepatocytes isolated from SRAKO (KO, n = 8) or WT (n = 8) littermates (7–8 weeks of age, chow diet) were analyzed by RT-qPCR. Protein expression of ATGL and SRAP was assessed by immunoblotting. (**b**) ATGL mediated ketone body production was measured in hepatocytes indicated in (**a**). (**c**) Endogenous SRA in Hepa1-6 cells was knocked down by lentiviral infection with shRNA against SRA (shSRA) or scrambled shRNA as control (shControl). Subsequent assays of mRNA and protein expression were performed 72 h after infection. (**d**) ATGL mediated ketone body production was measured in Hepa1-6 cells with shControl and shSRA knocked down at the same condition as (**c**). (**e**) Hepa1-6 cells were transfected with pSCT (Control), pSCT-SRA (SRA) or pSCT-SRAP-SDM1/7 (SRAP) expression vectors, and subsequent assays of RNA and protein expressions were performed 60 h after transfection. (**f**) ATGL-mediated ketone body production was measured in Hepa1-6 cells described in (**e**). The mRNA levels of genes were normalized to 36B4 expression. The data are presented as the mean ± SE, *p < 0.05.

We studied ATGL further and found that SRAKO increased liver ATGL mRNA (Fig. 1b) and protein (Fig. 1c) levels both under normal chow and high fat diet (HFD) feeding. ATGL is a major hepatic TAG lipase that plays an integral role in FFA partitioning and signaling to control energy metabolism^9^. Liver ATGL deficiency in mice causes progressive hepatic steatosis^8^, while hepatic overexpression of ATGL promotes FFA β-oxidation and ameliorates steatosis^7^. Therefore, ATGL expression induced by SRA deficiency may attenuate the hepatic steatosis due to HFD feeding. To confirm the regulation of ATGL by SRA, *in vitro* studies were performed in primary hepatocytes and Hepa1-6 cells. Figure 2a shows that, compared to wild type (WT), SRAKO hepatocytes have increased mRNA and protein levels of ATGL, which consequently induced higher ATGL enzyme activity (Supplementary Figure S2) and ATGL-mediated FFA β-oxidation assessed as ketone body production (Fig. 2b). Consistent with these data and the report that SRAKO mice have attenuated hepatic steatosis compared to WT littermates under the HFD feeding^29^, SRAKO hepatocytes are resistant to oleic acid-induced TAG accumulation (Supplementary Figure S3). In addition to the data in primary hepatocytes, similar results were found in the hepatocyte cell line Hapa1-6 following SRA knockdown (Fig. 2c,d). Conversely, overexpression of SRA RNA, but not the protein SRAP, inhibited ATGL mRNA and protein expression (Fig. 2e) and ketone body production in Hepa1-6 cells (Fig. 2f).

### Hepatic ATGL and SRA levels are inversely regulated in mice under fasting conditions

**Figure 3:**
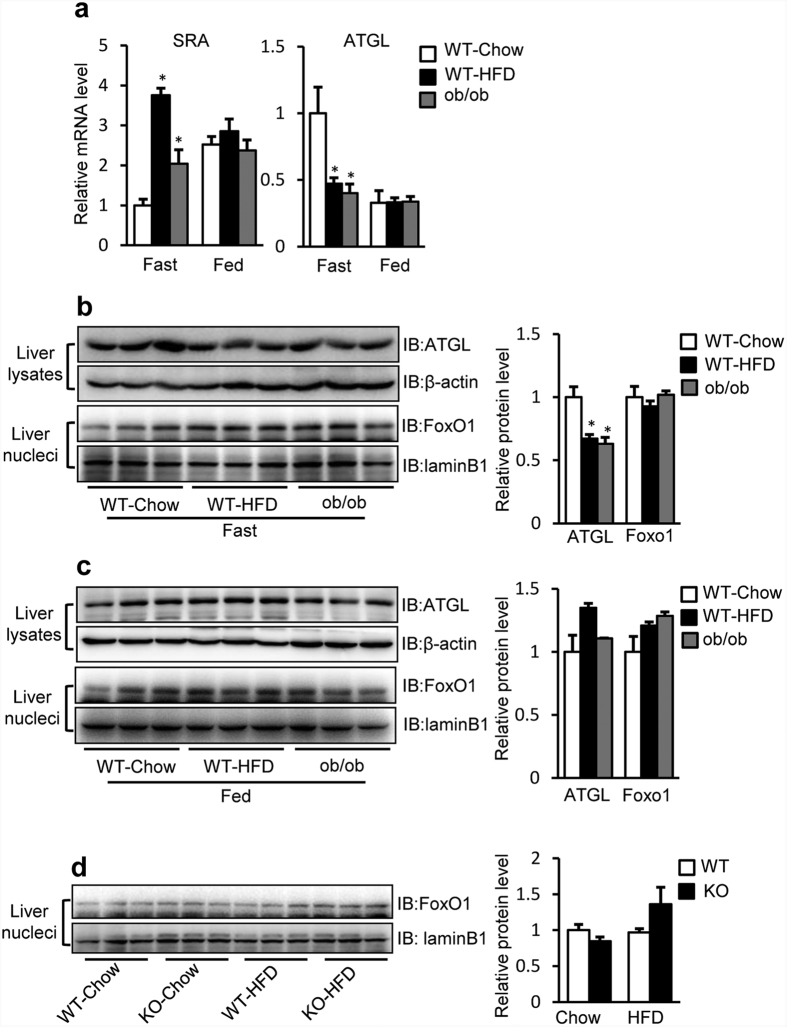
Hepatic levels of ATGL and SRA are inversely regulated in mice under fasting conditions. (**a**) SRA and ATGL mRNA levels in livers from WT-chow (n = 5) or WT-HFD (n = 6) and ob/ob (n = 8) mice (20 weeks of age) in either fasting or fed conditions were determined by RT-qPCR. The mRNA levels were normalized to 36B4 expression. Data are presented as mean ± SE and expressed as fold-change relative to WT-chow mice. (**b,c**) Liver or liver nuclear extracts from WT-chow, WT-HFD and ob/ob mice were immunoblotted with anti-FoxO1, anti-ATGL, anti-laminB1 and anti-β-actin antibodies. (**b**) Mice in fasting condition. (**c**) Mice in fed condition. (**d**) Liver nuclear extracts from WT or SRAKO (KO) mice fed with chow or HFD were immunoblotted with anti-FoxO1 and anti-laminB1 antibodies. (**b–d**) Right panels, quantification of bands in the immunoblots are shown. *p < 0.05.

In murine adipose tissue, ATGL mRNA expression transiently increases during fasting^32^. Next, we studied the effects of dietary manipulations on hepatic SRA and ATGL expression. We found that, under fasting conditions, liver SRA expression was increased to ~4 fold in WT-HFD mice and ~2 fold in leptin-deficient mice (ob/ob), compared to WT-Chow mice (Fig. 3a, left panel). In contrast, in the fed state, SRA levels were relatively higher and unaltered with HFD feeding or leptin deficiency.

In fasting conditions, hepatic ATGL mRNA levels were reduced in WT-HFD and ob/ob mice compared to WT-Chow mice, but no differences were found in fed conditions (Fig. 3a, right panel). Thus, under fasting, there is an inverse relationship between the mRNA levels of ATGL and SRA, consistent with the results in cultured cells (Fig. 2), implying that SRA may negatively regulate the transcription of ATGL. Reduced liver ATGL protein levels were also seen in WT-HFD and ob/ob mice compared to WT-Chow mice in fasting but not fed conditions (Fig. 3b,c). However, neither in fasting nor in fed conditions, could we find any differences in hepatic nuclear FoxO1 (Fig. 3b,c), the upstream transcription factor regulating ATGL expression previously shown in adipocytes^33^. Furthermore, SRA deficiency did not affect the nuclear FoxO1 protein content in liver (Fig. 3d). These results suggest that SRA regulates the expression of ATGL by a mechanism not involving the level change of nuclear FoxO1.

### SRA inhibits the transactivation of ATGL promoter via FoxO1

**Figure 4:**
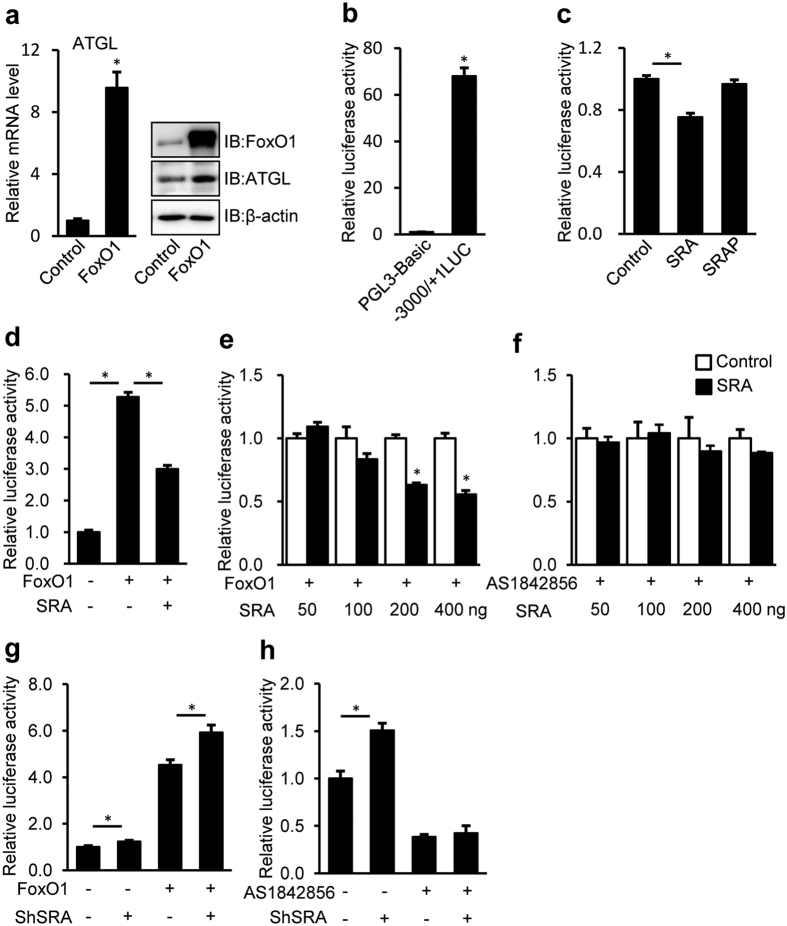
FoxO1 regulates ATGL expression and SRA inhibits FoxO1-mediated ATGL promoter driven luciferase activity. (**a**) Hepa1-6 cells were transfected with FoxO1 or control expression vector (pcDNA3.0). 60 h after transfection, ATGL mRNA expression (normalized to 36B4 expression) was measured by RT-qPCR (left panel) and cell extracts were immunoblotted with anti-FoxO1, anti-ATGL and anti-β-actin antibodies (right panel). (**b**) HepG2 cells were transfected with either pGL3-Basic empty vector (200 ng) or −3000/+ 1-LUC ATGL construct (200 ng) plus pRL-TK-*Renilla* (10 ng). (**c**) pSCT (Control, 200 ng), pSCT-SRA (SRA, 200 ng) or pSCT-SRAP-SDM1/7 (SRAP, 200 ng) were transfected with −3000/+ 1 LUC ATGL (200 ng) plus pRL-TK-*Renilla* (10 ng) into HepG2 cells. (**d**) −3000/+ 1 LUC ATGL (200 ng) plus pRL-TK-*Renilla* (10 ng) was transfected into HepG2 cells without or with FoxO1 (50 ng) and pSCT-SRA (SRA, 200 ng). (**e**) FoxO1 (50 ng) expression vector and increasing dose of pSCT-SRA as indicated were transfected with −3000/+ 1 LUC ATGL (200 ng) plus pRL-TK-*Renilla* (10 ng) into HepG2 cells. (**f**) Increasing doses of pSCT-SRA as indicated were transfected with −3000/+ 1 LUC ATGL (200 ng) plus pRL-TK-*Renilla* (10 ng) into HepG2 cells. Cells were treated with AS1842856 (1 μM) for 60 h after transfection. (**g,h**) Endogenous SRA in Hepa1-6 cells was knocked down by lentiviral infection with shRNA against SRA (shSRA) or scrambled shRNA as control (shControl). Subsequent transfection and luciferase assays were performed 12 h and 72 h after infection, respectively. (**g**) Hepa1-6 cells were transfected with −3000/+ 1 LUC construct (200 ng) plus pRL-TK-*Renilla* (10 ng) without or with FoxO1 (50 ng). (**h**) Hepa1-6 cells were cotransfected with −3000/+ 1 LUC ATGL (200 ng) plus pRL-TK-*Renilla* (10 ng) without or with AS1842856 (1 μM) treatment for 60 h after transfection. ATGL promoter driven luciferase activity were normalized to *Renilla* luciferase activity, presented as mean ± SE. Data are expressed as fold-change relative to the level of control. *p < 0.05.

It is well established that FoxO1 has binding sites in the −3 kb promoter region of the ATGL gene and upregulates ATGL transcription in adipocytes^33^. FoxO1 regulates both hepatic glucose production and lipid metabolism^34^,^35^. Overexpression of FoxO1 upregulates both the mRNA and protein levels of ATGL in hepa1-6 cells (Fig. 4a), suggesting that ATGL transcription is directly regulated by FoxO1 in hepatocytes. We utilized a 3 kb ATGL promoter luciferase reporter vector to assess this further, and found this vector showed 75 fold higher luciferase activity than the pGL3-Basic control (Fig. 4b). Cotransfection of a plasmid coding SRA RNA but not the protein SRAP inhibited ATGL promoter driven luciferase by ~25% (Fig. 4c). To confirm whether SRA exerts its inhibitory effect via FoxO1, FoxO1 and SRA were cotransfected with the luciferase system (Fig. 4d). As expected, FoxO1 itself increased ATGL promoter driven luciferase to ~5 fold, but cotransfection of SRA repressed this FoxO1 mediated effect (Fig. 4d). Furthermore, the ability of SRA to inhibit FoxO1 mediated luciferase activity was dose-dependent (Fig. 4e). In addition, the FoxO1 inhibitor AS1842856 (1 μM) induced the constant suppression of FoxO1 activity (Supplementary Figure S4a), and diminished the inhibitory effect of SRA on ATGL promoter activity (Fig. 4f). In contrast to the overexpression of SRA, SRA knockdown increased ATGL promoter activity (Fig. 4g,h), which was prevented by AS1842856 (Fig. 4h). Taken together, these data indicate that SRA regulates ATGL expression by inhibiting the ability of FoxO1 to promote ATGL transcription.

### The inhibitory effect of SRA on ATGL transcription is insulin independent

**Figure 5:**
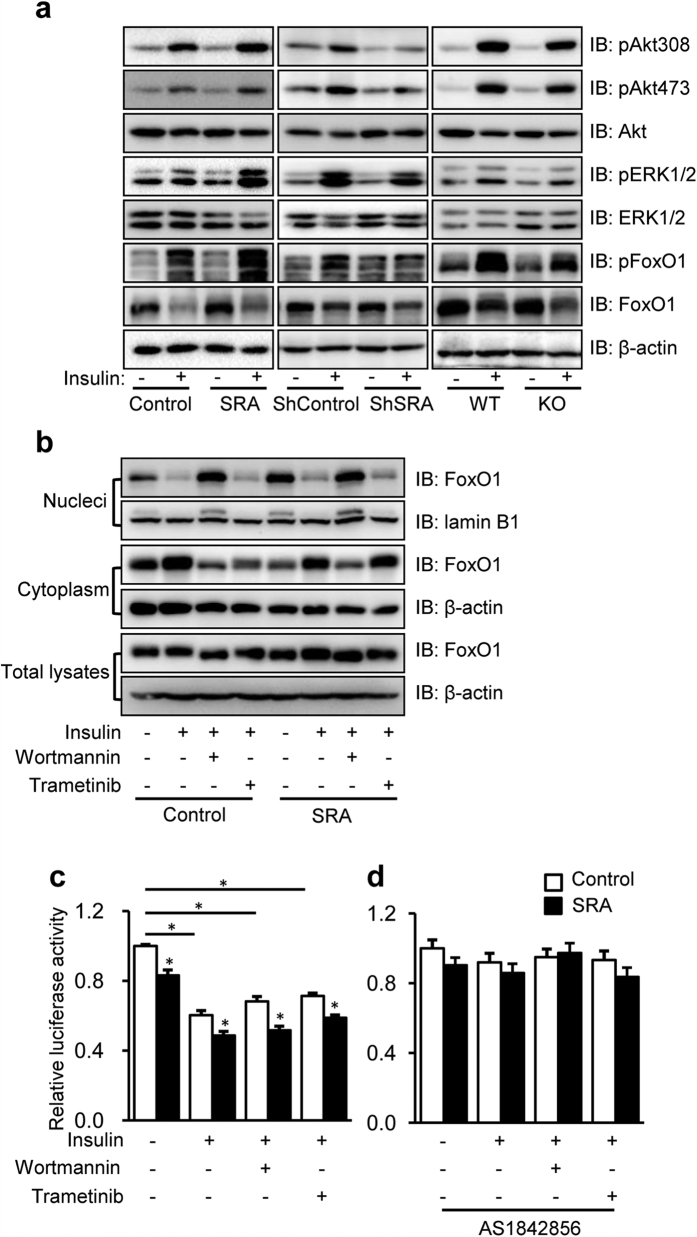
SRA promotes insulin-simulated phosphorylation of Akt, ERK1/2 and FoxO1, and inhibits ATGL transcription in an insulin-independent manner. (**a**) Left panel: Hepa1-6 cells were transfected with pSCT (Control) or pSCT-SRA (SRA) expression vectors and then cultured for 60 h; middle panel: Hepa1-6 cells were infected with lentivirus of scrambled shRNA (ShContronl) or shRNA against SRA (ShSRA) and then cultured for 72 h; right panel: Primary hepatocytes were isolated from SRAKO mice (KO) or WT littermates and cultured for 16 h. Cells were serum-starved for 5 h followed by treatment with or without insulin (10 nM) for 5 min. Afterwards, cell extracts were immunoblotted with the indicated antibodies. (**b**) Hepa1-6 cells were transfected with SRA or Control plasmids and then cultured for 60 h. After 5 h serum-starvation, cells were pretreated by MG132 (50 μM) with or without wortmannin (1 μM) or trametinib (1 μM) for 30 min, and finally treated with or without insulin (10 nM) for 5 min before harvesting. Then, proteins extracted from the nucleus, cytoplasm or cell lysates were immunoblotted with indicated antibodies. (**c**,**d**) HepG2 cells were cotransfected by −3000/+ 1 LUC ATGL (200 ng) plus pRL-TK-*Renilla* (10 ng) with either pSCT-SRA or control expression vectors (200 ng). Cells were treated without (**c**) or with (**d**) AS1842856 (1 μM) immediately after transfection. 36 h later, cells were serum starved and treated with wortmannin (1 μM) or trametinib (1 μM) plus insulin (10 nM) for further 24 h-culture. ATGL promoter driven luciferase activity were normalized to *Renilla* luciferase activity and presented as mean ± SE. Data are expressed as fold-change relative to the level of control. *p < 0.05.

In adipocytes, insulin induces shuttling of FoxO1 from the nucleus to the cytoplasm and thereby inhibits FoxO1’s binding to the ATGL promoter and ATGL transcription^33^. This inhibitory effect of insulin is likely through PI3K/Akt- and MEK/ERK-mediated phosphorylation of FoxO1^36^. In addition, insulin signaling via Akt-PI3K also promotes proteasome-dependent degradation of FoxO1^37^,^38^. Given our previous studies indicating that SRA enhances phosphorylation of Akt and its downstream target FoxO1 in response to insulin in differentiated adipocytes^27^,^28^, we examined whether SRA affects phosphorylation events in the downstream insulin pathway of hepatocytes. As shown in Fig. 5a (left panel), overexpression of SRA enhanced insulin-stimulated phosphorylation of Akt, Erk1/2 and FoxO1 in Hepa1-6 cells, similar to our previous findings in adipocytes. In contrast, loss of endogenous SRA by shRNA against SRA in Hepa1-6 cells (Fig. 5a middle panel) or in isolated hepatocytes from SRAKO mice (Fig. 5a right panel) decreased the phosphoryaltion of Akt, FoxO1, and Erk1/2. Interestingly, insulin also decreased the total FoxO1 protein content (Fig. 5a), which is likely due to protein degradation^37^,^38^. To assess whether SRA affects FoxO1 nuclear export in response to insulin, we pretreated Hepa1-6 cells with the proteasome inhibitor MG132, followed by treatment with or without insulin in combination with or without the PI3K/Akt inhibitor wortmannin or the MEK/ERK inhibitor trametinib (Fig. 5b). Unexpectedly, we found that SRA overexpression increased FoxO1 nuclear retention by 60% in the absence of insulin, but insulin eliminated this effect and decreased nuclear FoxO1 to low levels. The PI3K/Akt inhibitor wortmannin blocked the insulin-stimulated nuclear export of FoxO1, while the MEK/ERK inhibitor trametinib had no effect. Overall, these data indicate that the mechanism by which SRA suppresses FoxO1 activity does not involve enhanced nuclear export of FoxO1.

Reporter gene assays demonstrated that insulin reduced ATGL promoter driven luciferase activity, and wortmannin or trametinib did not completely reverse this inhibition (Fig. 5c). Overexpression of SRA induced similar proportional decreases in ATGL transcription under the different treatments above. However, the FoxO1 inhibitor AS1842856 eliminated the suppressive effects of SRA and insulin on ATGL promoter activity (Fig. 5d). These data suggest that the inhibitory effects of insulin and SRA on ATGL transcription are based on FoxO1, but SRA functions independent of insulin signaling.

### SRA inhibits transactivation of the ATGL promoter via PPARγ

**Figure 6:**
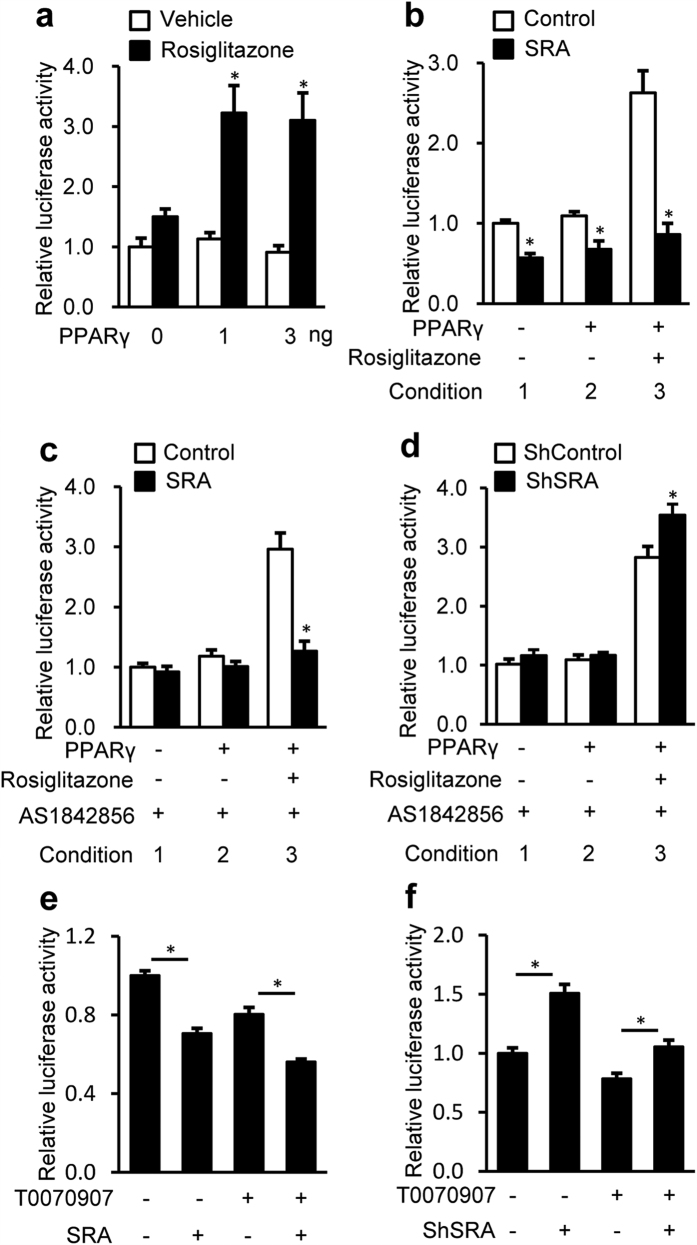
SRA inhibits PPARγ-mediated ATGL promotor driven luciferase activity. Cells were transfected with −3000/+ 1-LUC ATGL construct (200 ng) and pRL-TK-*Renilla* (10 ng). (**a–d**) PPARγ expression vector was cotransfected. 36 h after transfection, cells were treated with rosiglitazone (1 μM) for 24 h before harvesting. (**a**) HepG2 cells were transfected by PPARγ expression vector with the dose indicated. (**b**) pSCT-SRA or control expression vector (200 ng) plus PPARγ expression vector (3 ng) were transfected into HepG2 cells. (**c**) Similar to (**b**), except with AS1842856 (1 μM) treatment immediately after transfection. (**d**) Hepa1-6 cells were infected with shRNA against SRA (shSRA) or scrambled shRNA as control (shControl). 12 h later, PPARγ expression vector (3 ng) was transfected into Hepa1-6 cells. AS1842856 (1 μM) treatment was started immediately after transfection. Subsequent luciferase assays were performed 72 h after infection. (**e**) HepG2 cells were transfected by pSCT-SRA or control expression vector (200 ng), followed by T0070907 (0.4 μM) treatment immediately after transfection. 60 h later, cells were harvested for luciferase assay. (**f**) Hepa1-6 cells were infected with shRNA against SRA (shSRA) or scrambled shRNA as control (shControl). 12 h later, cells were treated with T0070907 (0.4 μM) for 60 h before harvesting. ATGL promoter driven luciferase activity were normalized to *Renilla* luciferase activity and presented as mean ± SE. Data are expressed as fold-change relative to the level of control. *p < 0.05.

**Figure 7:**
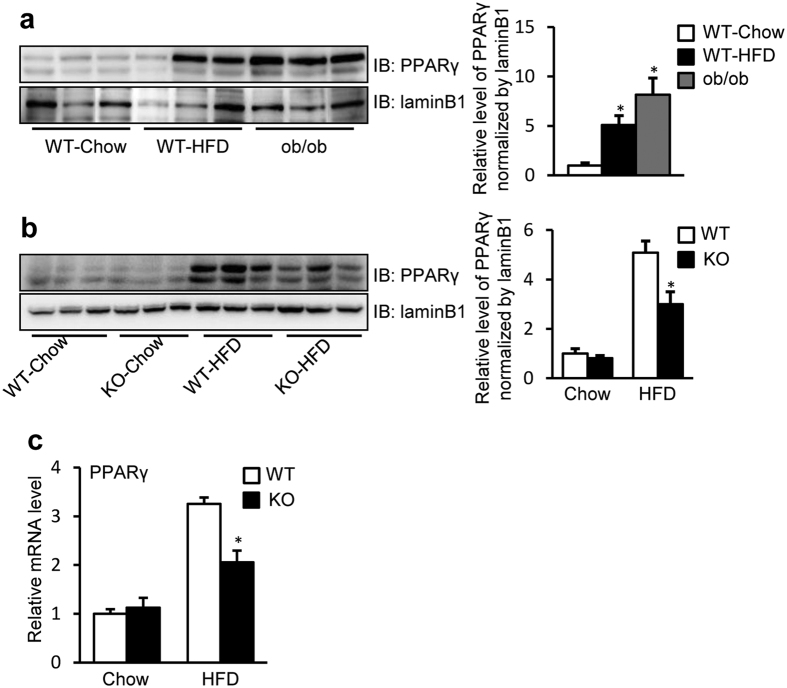
SRA promotes the expression of PPARγ. Animals were fasted for 16 h before sacrifice. (**a**) Liver nuclear extracts from male WT mice fed with chow diet or HFD, and ob/ob mice fed with chow diet (20 weeks of age), were immunoblotted with anti-PPARγ or anti-laminB1 antibodies. (**b**) Liver nuclear extracts from male WT or SRAKO (KO) mice fed with chow diet or HFD (20 weeks of age), were immunoblotted with anti-PPARγ and anti-laminB1 antibodies. (**c**) mRNA levels of PPARγ in livers of male SRAKO (KO, n = 8) or WT (n = 8) littermates (20 weeks of age) fed with normal chow or HFD. Quantification of bands and their relative intensities are shown in the right panel. Data are presented as mean ± SE and expressed as fold-change relative to the level of chow-fed WT mice. *p < 0.05.

In addition to FoxO1, PPARγ also has binding sites in the −3 kb ATGL promoter and induces ATGL transcription in adipocytes^39^. Despite its lower level in liver than that in adipose tissue, PPARγ significantly affects hepatic lipid metabolism^40^. Thus, it was important to investigate the effects of PPARγ on ATGL expression in hepatocytes, as well as the influence of SRA in the action of PPARγ. We found that, in the absence of PPARγ ligand rosiglitazone, overexpression of PPARγ has no effect on ATGL promoter driven luciferase activity in the HepG2 cells (Fig. 6a). However, in the presence of rosiglitazone, PPARγ increased ATGL promoter activity to ~3 fold. Next, we found that forced expression of SRA blocked the ability of PPARγ/rosiglitazone to induce ATGL promoter activity (Fig. 6b, conditions 3). To eliminate the potential influence of FoxO1, its inhibitor AS1842856 was used (Fig. 6c,d). Thus, the inhibitory effect of SRA in the absence of rosiglitazone was lost (Fig. 6c, conditions 1–2, compare with Fig. 6b conditions 1–2), but SRA still reduced ATGL promoter activity induced by rosiglitazone-bound PPARγ (Fig. 6c, condition 3). Conversely, SRA knockdown slightly enhanced ATGL promoter activity mediated by PPARγ/rosiglitazone (Fig. 6d, condition 3), but had no effect without rosiglitazone treatment when AS1842856 was present (Fig. 6d, conditions 1–2). These data indicate that SRA inhibits the inductive activity of PPARγ on the ATGL promoter when a PPARγ agonist is present, and this inhibition is independent of FoxO1. In preliminary studies we found that the PPARγ antagonist T0070907 exerts its maximal inhibitory effect on ATGL promoter activity at 0.4 μM (Supplementary Figure S4b), but its inhibitory potency was somewhat weaker than that of AS1842856 (Supplementary Figure S4a). We found that 0.4 μM T0070907 showed no effect on the ability of exogenous SRA to inhibit ATGL promoter activity (Fig. 6e), and only marginally impaired the ability of SRA knockdown to increase the ATGL promoter activity (Fig. 6f). This contrasts with AS1842856, which eliminated the effects of overexpressed SRA and SRA knockdown (Fig. 4f,h). Together, these data indicate that FoxO1, rather than PPARγ, is the dominant regulator of ATGL transcription in the absence of an exogenous PPARγ agonist. In the livers of mice with dietary obesity (HFD) or genetic obesity (ob/ob), PPARγ protein levels are dramatically increased (Fig. 7a), but this increment did not elevate the expression of ATGL (Fig. 3a,b). We reason that the potential stimulative effect of increased PPARγ expression may be counteracted by the inhibitory effect of increased SRA expression. Interestingly, the elevated mRNA and protein levels of hepatic PPARγ by HFD are substantially decreased in SRA deficiency (Fig. 7b,c). Further study will be needed to determine whether this reduction of PPARγ is due to the loss of SRA itself or is a consequence of reduced hepatic steatosis via SRA deficiency.

## Discussion

The mammalian genome encodes a large number of lncRNAs, which are defined as transcripts longer than 200 nt that lack any coding potential^41^,^42^. The physiological function of the vast majority of lncRNAs remains elusive^41^,^43^. SRA was originally characterized as non-coding RNA with a 680 nt core sequence^15^. Most experiments using transiently transfected reporter gene assays indicate that SRA coactivates multiple nuclear receptors (NRs) and the non-NR transcription factor by forming complexes with coactivator proteins such as SRC-1 and DEAD-box proteins p68/p72^15^,^18^,^20^,^44^,^45^. Our recent findings indicate that SRA plays multiple roles in the regulation of adipogenesis and insulin sensitivity in adipocytes^27^,^28^. Importantly, we have generated global SRA knockout mice, which are resistant to HFD-induced obesity with decreased fat mass and attenuated hepatic steatosis^29^, suggesting that SRA plays an important role not only in adipose tissue but also in liver.

ATGL is the major hepatic TAG hydrolase driving hepatic FFA oxidation^8^,^9^. It has a higher substrate specificity for TAG than hormone sensitive lipase (HSL), which previously was thought to be the predominant TAG hydrolase^46^. In the present study, we found that SRAKO upregulated the mRNA and protein levels of hepatic ATGL under both HFD and normal chow feeding (Fig. 1). Furthermore, ATGL expression was induced in SRAKO hepatocytes (Fig. 2a), resulting in increased ATGL enzyme activity in liver (Supplementary Figure S2) and activated hepatocyte ketone body production (Fig. 2b), a product and indicator of FFA β-oxidation. Similar results also were found in the hepatocyte cell line, Hepa1-6, with knockdown of endogenous SRA (Fig. 2c,d). In contrast, forced expression of SRA inhibited the expression of ATGL and reduced FFA β-oxidation (Fig. 2e,f). Importantly, we demonstrate that SRA is induced in the livers of obesity after fasting (Fig. 3a), and that the hepatic levels of SRA and ATGL are inversely correlated in WT-Chow, WT-HFD and ob/ob mice (Fig. 3a,b). Thus, SRA levels fluctuate in response to energy levels and metabolic states, which lead to the inverse expression of liver ATGL.

The transcription factor FoxO1 induces ATGL expression in adipocytes^33^ and hepatocytes (Fig. 4a). A combination of approaches including genetic knockout *in vivo*, and overexpression, knockdown, and inhibitor studies in cultured cells indicate that SRA inhibits ATGL transcription by interfering with the transcriptional activity of FoxO1 (Figs 1, 3 and 4).

In adipocytes, insulin induces the shuttling of FoxO1 from the nucleus to the cytoplasm and thereby inhibits the transcription of the ATGL^33^. Previous studies in adipocytes^27^,^28^ and the present study in hepatocytes (Fig. 5a) show that SRA stimulates insulin-induced phosphorylation of Akt, Erk1/2 and their downstream target FoxO1, which seems contradictory to our previous data that SRAKO attenuated the insulin resistance induced by HFD^29^. However, the improved insulin sensitivity in SRAKO mice is likely secondary to the attenuation of hepatic steatosis and obesity. Insulin and SRA both inhibit FoxO1-mediated transcription of ATGL, but the mechanisms differ since only insulin induces nuclear export of FoxO1 (Fig. 5b), and the repressive effect of SRA is insulin-independent. Perhaps, the effect of SRA on promoting insulin signaling is too little to modulate FoxO1 nuclear localization, at least under the conditions of present study.

PPARγ, another transcription factor upregulating ATGL transcription in adipocytes^39^,^47^, is confirmed to have a similar effect in hepatocytes when activated by a PPARγ ligand (Fig. 6a). However, the elevated ATGL transcription by ligand-activated PPARγ was reduced to basal levels by SRA (Fig. 6b), an effect that is independent of FoxO1 (Fig. 6c). Despite the potential importance of the SRA-PPARγ-ATGL pathway, studies using FoxO1 and PPARγ inhibitors indicate that, in the absence of a PPARγ ligand, SRA mediates its repressive effects on ATGL transcription via FoxO1, not PPARγ (Figs 4 and 6).

The molecular mechanism underlying SRA functioning as a repressor in FoxO1 and PPARγ-mediated ATGL transcription remains to be fully elucidated. Previous studies indicate that SRA can function as an RNA coactivator by forming a complex with protein coactivators such as SRC-1^15^, and that it may function as a scaffolding molecule in these complexes^19^,^20^,^48^. However, SRA also has a repressive function and interacts with NR corepressors SHARP^49^ and SLIRP^50^, and acts as scaffold of repressive complexes containing HP1, LSD1, HDAC1/2 and CoREST to silence progesterone receptor regulated genes in the absence of hormones^51^. In addition, SRA can form complexes with trithorax groups and polycomb repressive complex 2 complexes to deliver either activating or repressive histone modifications^52^. Therefore, SRA can exert both coactivating and repressing functions dependent on its context. We hypothesize that, in liver, SRA functions as a scaffold to recruit a repressive complex to suppress FoxO1 and PPARγ-mediated ATGL transcription.

In summary, the present study shows that the hepatic expression of SRA and ATGL is inversely correlated, and the loss of SRA in the mouse liver or hepatocytes upregulates the expression of ATGL primarily by promoting the inductive effect of FoxO1, which may lead to increased FFA β-oxidation and confer protection against hepatic steatosis in diet-induced obesity.

## Methods

### Animals and diets

SRAKO mice were generated as described previously^29^. Leptin-deficient mice (ob/ob) were purchased from the Model Animal Research Center of Nanjing University, China. The mice were housed in a pathogen-free barrier facility with a 12 h light/dark cycle and were given free access to food and water. At the age of 8 weeks, SRAKO mice (KO) and wild type littermates (WT) were fed either a standard laboratory rodent chow diet (Chow) (Xietong Organism, Nanjing, China) or a high fat diet (HFD, 60% fat, 20% carbohydrate, 20% protein; Research Diets, New Brunswick, USA) for 12 weeks. ob/ob mice were fed chow diet until 20 weeks old. Mice were sacrificed during the light phase after food deprivation for 16 h or without fasting. Tissues were isolated immediately, weighed and stored in liquid nitrogen. All animal husbandry and animal experiments complied with the guidelines of the Nanjing Medical University’s Regulations of Animal Experiments and were approved by the Animal Experiment Committee of the Nanjing Medical University.

### RNA extraction and quantitative real-time PCR

Total RNA was extracted from livers or cells using RNAiso Plus (Takara Bio Inc., Dalian, China) and was reverse-transcribed using M-MMLV Reverse Transcriptase with RNasin Ribonuclease Inhibitors (Promega Biotech Co., Ltd, Beijing, China) according to the manufacturer’s protocols. qPCR was performed using SYBR Premix Master Mix (Thermo Scientific Inc., Shanghai, China). Primer sequences for Pparα, Mcad, Lcad, Cpt1α, Cpt2, Cd36, Fatp1, L-fabp, Acsl1,Acox1, Acaa1b, Acaa2, Atgl, Hsl, Acly, Fasn, Acc1, Acc2, Scd1, Mtgpat1, Dgat1, Dgat2, Apob, Mttp, Apoc2, Apoc3, Vldlr, and Sra are summarized in Supplementary Table S1. The expression levels of mRNA were normalized to those of ribosomal protein, large, P0 (36B4) mRNA.

### Immunoblotting

Proteins from liver lysates, cell lysates, cell nucleus and cell cytoplasm were extracted by previous methods^53^ or kits (Beyotime Biotechnology, Shanghai, China). Primary antibodies were used at the dilution of 1:1000. Anti-phospho-FoxO1 (#9461), FoxO1 (#2880), phospho-ERK1/2 (#4370), ERK1/2 (#4695), phospho-Akt (Thr308) (#9275), phospho-Akt (Ser473) (#9271), Akt (#4685), ATGL (#2439), PPARγ (#2435), lamin B1 (#13435), β-actin (#3700) and horseradish peroxidase-conjugated anti-mouse or rabbit IgG were purchased from Cell Signaling Technology. Anti-SRAP antibody (#A300-743A) was purchased from Bethyl Laboratory, Inc.

### Cell culture and treatment

HepG2 human hepatocarcinoma cells and Hepa1-6 mouse hepatocarcinoma cells were cultured in Dulbecco’s modified Eagle’s medium (DMEM; Invitrogen), supplemented with 100 mg/ml streptomycin sulphate (Sigma–Aldrich), 100 U/ml penicillin G (Sigma–Aldrich) and 10% (v/v) fetal bovine serum (FBS; Invitrogen), and maintained at 37 °C in an atmosphere of 5% CO_2_ and 95% air. Primary hepatocytes were isolated from wild type or SRAKO mice and grown in William’s medium E (Sigma–Aldrich) with FBS and antibiotics as described previously^54^. Cells were serum-starved for 5 h followed by treatment with or without insulin (10 nM) for 5 min. Before insulin treatment, cells were pretreated with MG132 (Sigma–Aldrich, 50 μM), wortmannin (Sigma–Aldrich, 1 μM) or trametinib (MedChem Express, 1 μM) for 30 min.

### Plasmids, transfection, retroviral infection and reporter gene assays

PPARγ was expressed in mammalian cells from the vector pFlag-CMV-7.1 (Sigma) as described previously^28^. The −3000 to +1 promoter region of mouse gene *Pnpla2* (ATGL) was amplified by PCR from mouse liver genomic DNA using primers 5′-CACAACGCGTCCAGCCCTGTTCTTGGCACCAGCATC-3′ and 5′-CACACTCGAGGGTGTTTTAGTGCGGGGTGAGGGCG-3′ with Mlu I and Xho I overhangs. The PCR inserts were then ligated to Mlu I and Xho I sites in the pGL3-Basic vector to obtain the reporter construct pGL3-3000/+ 1LUC. The human SRA expression vector pSCT-SRA was kindly provided by R. Lanz (Baylor College of Medicine, Houston, TX)^15^. The plasmid pSCT-SRA (denoted SRA only), contains the human SRA RNA core sequence and hence expresses SRA but not SRAP. As described previously^27^, pSCT-SRAP-SDM1/7 (denoted as SRAP only), with a series of silent mutations in SRA RNA stem loops 1 and 7 produces wild type SRAP and dysfunctional SRA. The human pcDNA-V5-tagged FoxO1 expression vector was kindly provided by Robert Tjian^54^. Transient transfections and luciferase reporter gene assays were performed as described previously^20^. Hepa1-6 cells were transfected with expression vectors in 6-well plates for RT-qPCR or immunoblotting. HepG2 cells were transfected with expression vectors or luciferase reporter plasmids plus the internal control vector pRL-TK-*Renilla* for luciferase assays in 24-well plates. After overnight culture, cells were incubated for 4 h in serum-free DMEM containing DNA-polyethylenimine (Sigma, 1 mg/ml) complex and then grown in DMEM supplemented with 10% FBS for 60 h. Luciferase activity was measured using the Dual-Luciferase reporter assay system (Promega, Madison, WI) and normalized to *Renilla* luciferase values.

### Gene silencing by short hairpin RNA (shRNA)

According to previous methods^27^, endogenous mouse SRA in Hepa1-6 cells was knocked down by infection with a lentivirus expressing shRNA directed against SRA generated in 293T cells, supplemented with 8 mg/ml Polybrene (Sigma) after overnight attachment. Infection was repeated at intervals of 8 to 12 h. Cells were subjected to experiments 72 h after the second infection.

### ATGL mediated FFA β-oxidation assays

Hepatocytes or Hepa1-6 cells were cultured in complete medium with oleic acid (100 μM) for 16 h. Then, cells were incubated in serum free, phenol red free and glucose free DMEM (Sigma) for 4 h with or without (R)-Bromoenol lactone (25 μM, Cayman Chemical company, Ann Arbor, USA). Ketone bodies, the products of FFA β-oxidation liberated into the media were assayed by D-3-hydroxybutyrate assay kit (Megazyme Internatioanl Ireland, Bray, Ireland). ATGL-mediated FFA β-oxidation was calculated as the difference of D-3-hydroxybutyrate production between cells treated with or without the ATGL specific inhibitor, (R)-bromoenol lactone, normalized to cell protein level, i.e. the portion of D-3-hydroxybutyrate production suppressible with (R)-bromoenol lactone.

### ATGL activity assays

TAG hydrolase activity in liver lysates was assayed with a TAG hydrolase assay kit (Jiancheng Bioengineering Institute, Nanjing, China) and normalized to liver lysate protein levels. ATGL activity was calculated as the difference of TAG hydrolase activity in the presence and absence of (R)-bromoenol lactone (25 μM), i.e. the portion of TAG hydrolase activity suppressible with (R)-bromoenol lactone.

### TAG assay in hepatocytes

Hepatocytes were homogenized in 0.1 M HCl and extracted by chloroform-methanol (2:1). The organic phase was evaporated to dry. Lipid residues were dissolved in isopropanol and measured using the TAG assay kit (GPO-PAP; Dongou Bioengineering Co. Ltd, Wenzhou, China). TAG levels were normalized to protein levels of hepatocytes.

### Statistical analysis

Results are expressed as mean ± SE. Data between groups were analyzed by Student’s t-test or one-way ANOVA followed by Bonferroni–Dunn multiple comparison. Differences were considered significant at *P* < 0.05.

## Additional Information

**How to cite this article**: Chen, G. *et al*. LncRNA SRA promotes hepatic steatosis through repressing the expression of adipose triglyceride lipase (ATGL). *Sci. Rep.* **6**, 35531; doi: 10.1038/srep35531 (2016).

## Supplementary Material

Supplementary Information

## References

[b1] Marchesini G. . Nonalcoholic fatty liver disease: a feature of the metabolic syndrome. Diabetes 50, 1844–1850 (2001). 11473047 10.2337/diabetes.50.8.1844

[b2] Youssef W. & Mccullough A. J. Diabetes mellitus, obesity, and hepatic steatosis. Seminars in Gastrointestinal Disease 13, 17–30 (2002). 11944630

[b3] Rinella M. E. Nonalcoholic fatty liver disease: a systematic review. Jama the Journal of the American Medical Association 313, 2263–2273 (2015). 26057287 10.1001/jama.2015.5370

[b4] Shaker M., Tabbaa A., Albeldawi M. & Alkhouri N. Liver transplantation for nonalcoholic fatty liver disease: New challenges and new opportunities. World Journal of Gastroenterology 20, 5320–5330 (2014). 24833862 10.3748/wjg.v20.i18.5320 PMC4017047

[b5] Ruhl C. E. & Everhart J. E. Fatty liver indices in the multiethnic United States National Health and Nutrition Examination Survey. Alimentary Pharmacology & Therapeutics 41, 65–76 (2015). 25376360 10.1111/apt.13012

[b6] Goldberg I. J. & Ginsberg H. N. Ins and outs modulating hepatic triglyceride and development of nonalcoholic fatty liver disease. Gastroenterology 130, 1343–1346 (2006). 16618425 10.1053/j.gastro.2006.02.040

[b7] Reid B. N. . Hepatic overexpression of hormone-sensitive lipase and adipose triglyceride lipase promotes fatty acid oxidation, stimulates direct release of free fatty acids, and ameliorates steatosis. Journal of Biological Chemistry 283, 13087–13099 (2008). 18337240 10.1074/jbc.M800533200 PMC2442319

[b8] Wu J. W. . Deficiency of liver adipose triglyceride lipase in mice causes progressive hepatic steatosis † ‡. Hepatology 54, 122–132 (2011). 21465509 10.1002/hep.24338

[b9] Ong, K. T., Mashek, M. T., Bu, S. Y., Greenberg, A. S. & §, D. G. M. Adipose triglyceride lipase is a major hepatic lipase that regulates triacylglycerol turnover and fatty acid signaling and partitioning † ‡. Hepatology 53, 116–126 (2011). 20967758 10.1002/hep.24006 PMC3025059

[b10] Haemmerle G. . Defective lipolysis and altered energy metabolism in mice lacking adipose triglyceride lipase. Science 312, 734–737 (2006). 16675698 10.1126/science.1123965

[b11] Kienesberger P. C. . Adipose triglyceride lipase deficiency causes tissue-specific changes in insulin signaling. Journal of Biological Chemistry 284, 30218–30229 (2009). 19723629 10.1074/jbc.M109.047787 PMC2781577

[b12] Rinn J. L. & Chang H. Y. Genome regulation by long noncoding RNAs. Annual Review of Biochemistry 81, 145–166 (2012). 10.1146/annurev-biochem-051410-092902 PMC3858397 22663078

[b13] Wang X., Song X., Glass C. K. & Rosenfeld M. G. The long arm of long noncoding RNAs: roles as sensors regulating gene transcriptional programs. Cold Spring Harbor Perspectives in Biology 3, a003756–a003756 (2011). 20573714 10.1101/cshperspect.a003756 PMC3003465

[b14] Li P. . A Liver-Enriched Long Non-Coding RNA, lncLSTR, Regulates Systemic Lipid Metabolism in Mice. Cell metabolism 21, 455–467 (2015). 25738460 10.1016/j.cmet.2015.02.004 PMC4350020

[b15] Lanz R. B. . A Steroid Receptor Coactivator, SRA, Functions as an RNA and Is Present in an SRC-1 Complex. Cell 97, 17–27 (1999). 10199399 10.1016/s0092-8674(00)80711-4

[b16] Xu B. & Koenig R. J. An RNA-binding domain in the thyroid hormone receptor enhances transcriptional activation. Journal of Biological Chemistry 279, 33051–33056 (2004). 15180993 10.1074/jbc.M404930200

[b17] Zhao X. . Regulation of Nuclear Receptor Activity by a Pseudouridine Synthase through Posttranscriptional Modification of Steroid Receptor RNA Activator. Molecular Cell 15, 549–558 (2004). 15327771 10.1016/j.molcel.2004.06.044

[b18] Caretti G. . The RNA Helicases p68/p72 and the Noncoding RNA SRA Are Coregulators of MyoD and Skeletal Muscle Differentiation. Developmental Cell 11, 547–560 (2006). 17011493 10.1016/j.devcel.2006.08.003

[b19] Hubé F., Velasco G., Rollin J., Furling D. & Francastel C. Steroid receptor RNA activator protein binds to and counteracts SRA RNA-mediated activation of MyoD and muscle differentiation. Nucleic Acids Research 39, 513–525 (2011). 20855289 10.1093/nar/gkq833 PMC3025577

[b20] Xu B. . Dax-1 and steroid receptor RNA activator (SRA) function as transcriptional coactivators for steroidogenic factor 1 in steroidogenesis. Molecular & Cellular Biology 29, 1719–1734 (2009). 19188450 10.1128/MCB.01010-08 PMC2655620

[b21] Lanz R. B., Razani B., Goldberg A. D. & O’Malley B. W. Distinct RNA motifs are important for coactivation of steroid hormone receptors by steroid receptor RNA activator (SRA). Proceedings of the National Academy of Sciences 99, 16081–16086 (2002). 10.1073/pnas.192571399 PMC138568 12444263

[b22] Leygue E., Dotzlaw H., Watson P. H. & Murphy L. C. Expression of the steroid receptor RNA activator in human breast tumors. Cancer Research 59, 4190–4193 (1999). 10485452

[b23] Murphy L. C. . Altered expression of estrogen receptor coregulators during human breast tumorigenesis. Cancer Research 60, 6266–6271 (2000). 11103781

[b24] Friedrichs F. . HBEGF, SRA1, and IK: Three cosegregating genes as determinants of cardiomyopathy. Genome Research 19, 395–403 (2009). 19064678 10.1101/gr.076653.108 PMC2661798

[b25] E E. . Identification of new human coding steroid receptor RNA activator isoforms. Biochemical & Biophysical Research Communications 301, 509–515 (2003). 12565891 10.1016/s0006-291x(02)03070-x

[b26] Kawashima H. . A novel steroid receptor co-activator protein (SRAP) as an alternative form of steroid receptor RNA-activator gene: expression in prostate cancer cells and enhancement of androgen receptor activity. Biochemical Journal 369, 163–171 (2003). 12350225 10.1042/BJ20020743 PMC1223065

[b27] Xu B. . Multiple Roles for the Non-Coding RNA SRA in Regulation of Adipogenesis and Insulin Sensitivity. Plos One 5, e14199 (2010). 21152033 10.1371/journal.pone.0014199 PMC2996286

[b28] Liu S. . SRA Regulates Adipogenesis by Modulating p38/JNK Phosphorylation and Stimulating Insulin Receptor Gene Expression and Downstream Signaling. Plos One 9, e95416 (2014). 24743795 10.1371/journal.pone.0095416 PMC3990642

[b29] Liu S. . SRA gene knockout protects against diet-induced obesity and improves glucose tolerance. Journal of Biological Chemistry 289, 13000–13009 (2014). 24675075 10.1074/jbc.M114.564658 PMC4036315

[b30] Cortés V. A. & Fernández-Galilea M. Lipodystrophies: adipose tissue disorders with severe metabolic implications. Journal of Physiology & Biochemistry 71, 1–8 (2015). 25833179 10.1007/s13105-015-0404-1

[b31] Roman S. . Brown adipose tissue and novel therapeutic approaches to treat metabolic disorders. Translational Research 165, 464–479 (2015). 25433289 10.1016/j.trsl.2014.11.002

[b32] Villena J. A., Roy S., Sarkadi-Nagy E., Kim K. H. & Sul H. S. Desnutrin, an adipocyte gene encoding a novel patatin domain-containing protein, is induced by fasting and glucocorticoids: ectopic expression of desnutrin increases triglyceride hydrolysis. Journal of Biological Chemistry 279, 47066–47075 (2004). 15337759 10.1074/jbc.M403855200

[b33] Chakrabarti P. & Kandror K. V. FoxO1 controls insulin-dependent adipose triglyceride lipase (ATGL) expression and lipolysis in adipocytes. Journal of Biological Chemistry 284, 13296–13300 (2009). 19297333 10.1074/jbc.C800241200 PMC2679428

[b34] Cook J. R. . A Mutant Allele Encoding DNA Binding-Deficient FoxO1 Differentially Regulates Hepatic Glucose and Lipid Metabolism. Diabetes 64, 1951–1965 (2015). 25576059 10.2337/db14-1506 PMC4439558

[b35] Matsumoto M., Han S., Kitamura T. & Accili D. Dual role of transcription factor FoxO1 in controlling hepatic insulin sensitivity and lipid metabolism. Journal of Clinical Investigation 116, 2464–2472 (2006). 16906224 10.1172/JCI27047 PMC1533874

[b36] Lp V. D. H., Hoekman M. F. & Smidt M. P. The ins and outs of FoxO shuttling: mechanisms of FoxO translocation and transcriptional regulation. Biochemical Journal 380, 297–309 (2004). 15005655 10.1042/BJ20040167 PMC1224192

[b37] Huang H. & Tindall D. J. Regulation of FOXO protein stability via ubiquitination and proteasome degradation ☆. Biochimica Et Biophysica Acta 1813, 1961–1964 (2011). 21238503 10.1016/j.bbamcr.2011.01.007 PMC3110514

[b38] Matsuzaki H. & Fukamizu A. Insulin-induced phosphorylation of FKHR (Foxo1) targets to proteasomal degradation. Proceedings of the National Academy of Sciences of the United States of America 100, 11285–11290 (2003). 13679577 10.1073/pnas.1934283100 PMC208749

[b39] Kim J. Y., Tillison K., Lee J. H., Rearick D. A. & Smas C. M. The adipose tissue triglyceride lipase ATGL/PNPLA2 is downregulated by insulin and TNF-alpha in 3T3-L1 adipocytes and is a target for transactivation by PPARgamma. American Journal of Physiology Endocrinology & Metabolism 291, E115–E127 (2006). 16705060 10.1152/ajpendo.00317.2005

[b40] Matsusue K. . Liver-specific disruption of PPARgamma in leptin-deficient mice improves fatty liver but aggravates diabetic phenotypes. Journal of Clinical Investigation 111, 737–747 (2003). 12618528 10.1172/JCI17223 PMC151902

[b41] Derrien T. . The GENCODE v7 catalog of human long noncoding RNAs: analysis of their gene structure, evolution, and expression. Genome Research 22, 1775–1789 (2012). 22955988 10.1101/gr.132159.111 PMC3431493

[b42] Mitchell G. . Chromatin signature reveals over a thousand highly conserved large non-coding RNAs in mammals. Nature 458, 223–227 (2009). 19182780 10.1038/nature07672 PMC2754849

[b43] Guttman M. & Rinn J. L. Modular regulatory principles of large non-coding RNAs. Nature 482, 339–346 (2012). 22337053 10.1038/nature10887 PMC4197003

[b44] Watanabe M. . A subfamily of RNA-binding DEAD-box proteins acts as an estrogen receptor alpha coactivator through the N-terminal activation domain (AF-1) with an RNA coactivator, SRA. Embo Journal 20, 1341–1352 (2001). 11250900 10.1093/emboj/20.6.1341 PMC145523

[b45] Xu B. & Koenig R. J. Regulation of thyroid hormone receptor α2 RNA binding and subcellular localization by phosphorylation. Molecular & Cellular Endocrinology 245, 147–157 (2005). 16356627 10.1016/j.mce.2005.11.010

[b46] Zimmermann R. . Fat mobilization in adipose tissue is promoted by adipose triglyceride lipase. Science 306, 1383–1386 (2004). 15550674 10.1126/science.1100747

[b47] Kershaw E. E. . PPARγ regulates adipose triglyceride lipase in adipocytes *in vitro* and *in vivo*. American Journal of Physiology Endocrinology & Metabolism 293, 1736–1745 (2007). 10.1152/ajpendo.00122.2007 PMC2819189 17848638

[b48] Caretti G., Lei E. P. & Sartorelli V. The DEAD-Box p68/p72 Proteins and the Noncoding RNA Steroid Receptor Activator SRA: Eclectic Regulators of Disparate Biological Functions. Cell cycle 6, 1172–1176 (2007). 17495528 10.4161/cc.6.10.4228

[b49] Shi Y. . Sharp, an inducible cofactor that integrates nuclear receptor repression and activation. Genes & Development 15, 1140–1151 (2001). 11331609 10.1101/gad.871201 PMC312688

[b50] Hatchell E. C. . SLIRP, a Small SRA Binding Protein, Is a Nuclear Receptor Corepressor. Molecular Cell 22, 657–668 (2006). 16762838 10.1016/j.molcel.2006.05.024

[b51] Vicent G. P. . Unliganded progesterone receptor-mediated targeting of an RNA-containing repressive complex silences a subset of hormone-inducible genes. Genes & Development 27, 1179–1197 (2013). 23699411 10.1101/gad.215293.113 PMC3672650

[b52] Wongtrakoongate P., Riddick G., Fucharoen S. & Felsenfeld G. Association of the Long Non-coding RNA Steroid Receptor RNA Activator (SRA) with TrxG and PRC2 Complexes. PLoS genetics 11 (2015). 10.1371/journal.pgen.1005615 PMC4619771 26496121

[b53] Sheng L. . NF-κB-inducing kinase (NIK) promotes hyperglycemia and glucose intolerance in obesity by augmenting glucagon action. Nature Medicine 18, 943–949 (2012). 10.1038/nm.2756 PMC3766969 22581287

[b54] Puig O. & Tjian R. Transcriptional feedback control of insulin receptor by dFOXO/FOXO1. Genes & Development 19, 2435–2446 (2005). 16230533 10.1101/gad.1340505 PMC1257398

