# Supplementary material for: LncRNA SRA promotes hepatic steatosis through repressing the expression of adipose triglyceride lipase (ATGL)
Source: Sci Rep. 2016 Oct 19;6:35531. doi: 10.1038/srep35531 (PMC5069493; doi:10.1038/srep35531)

LncRNA SRA promotes hepatic steatosis through repressing expression of adipose triglyceride lipase (ATGL)

Gang Chen1# and Dongsheng Yu2#, Xue Nian2, Junyi Liu3, Ronald J. Koenig4, Bin Xu4* and Liang Sheng2*

1 Department of Hepatobiliary Surgery, The First Affiliated Hospital, Wenzhou Medical University, Wenzhou 325000, China

2 Department of Pharmacology, School of Basic Medical Science, Nanjing Medical University, 140 Hanzhong Rd., Nanjing, Jiangsu, 210029, China

3 Department of Chemical Biology, School of Pharmaceutical Sciences, Peking University, Beijing 100191, China

4 Department of Internal Medicine, Division of Metabolism, Endocrinology and Diabetes, University of Michigan Medical Center, Ann Arbor, MI 48109-5678

*Corresponding authors: Liang Sheng, PhD.
Department of Pharmacology, School of Basic Medical Science,
Nanjing Medical University,
140 Hanzhong Rd., Nanjing, Jiangsu, 210029, China
Email: lgsheng@njmu.edu.cn
Phone: +86 13913007736
Fax: +86 83237637

Bin Xu, Ph.D.
Department of Internal Medicine, Division of Metabolism, Endocrinology and Diabetes, University of Michigan Medical Center,
Ann Arbor, MI 48109-5678
Email: bxu@umich.edu
Phone: 734-647-2883
Fax: 734-936-6684

#These authors contributed equally to this work.

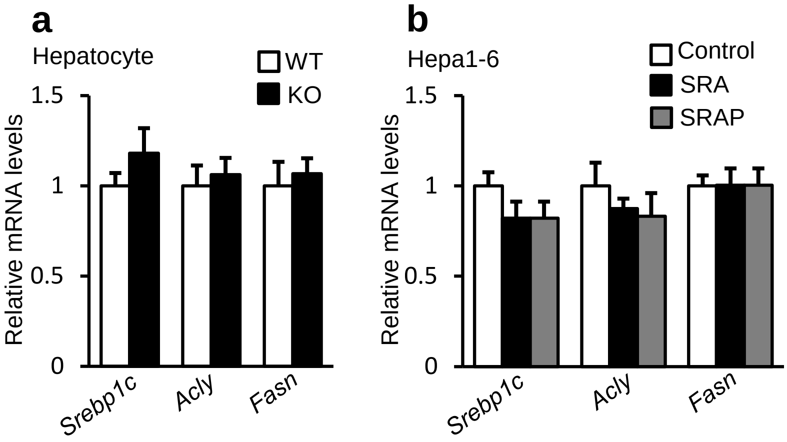

Supplementary Figure S1. Loss or overexpression of SRA does not affect the expression of Srebp1c, Acly or Fasn in hepatocytes. (a) The mRNA levels of Srebp1c, Acly and Fasn in primary hepatocytes isolated from SRAKO (KO, n=8) or WT (n=8) littermates (7-8 weeks of age, chow diet) were analyzed by RT-qPCR. (b) Hepa1-6 cells were transfected with pSCT (Control), pSCT-SRA (SRA) or pSCT-SRAP-SDM1/7 (SRAP) expression vectors, and subsequent assays were performed 60 h after transfection. The mRNA levels of Srebp1c, Acly and Fasn were analyzed by RT-qPCR and normalized to 36B4. The data are expressed as fold-change relative to control and presented as the mean ± SE. $^{*}p < 0.05$.

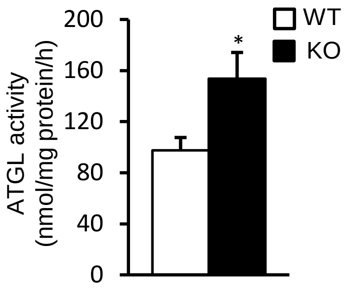

Supplementary Figure S2. Loss of SRA enhances ATGL activity in liver. Liver lysates from SRAKO (KO, n=8) or WT (n=8) mice (20 weeks of age, chow diet) were analyzed for TAG hydrolase activity normalized by liver lysate protein levels. ATGL activity was calculated as the difference between TAG hydrolase activity in the presence and absence of the ATGL inhibitor, (R)-bromoenol lactone (25 μM), i.e. TAG hydrolase activity suppressible with (R)-bromoenol lactone. The data are presented as the mean ± SE. *$p < 0.05$.

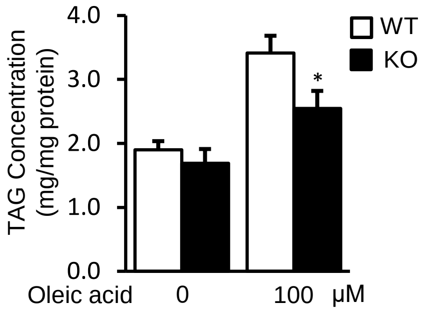

Supplementary Figure S3. Loss of SRA prevents the oleic acids induced TAG accumulation in hepatocytes. Hepatocytes isolated from SRAKO (KO, n=8) or WT (n=8) littermates (7-8 weeks of age, chow diet) were treated without or with oleic acids (100 μM) for 24h. TAG concentration was assayed and normalized to cellular protein. The data are presented as the mean ± SE. $^*p < 0.05$.

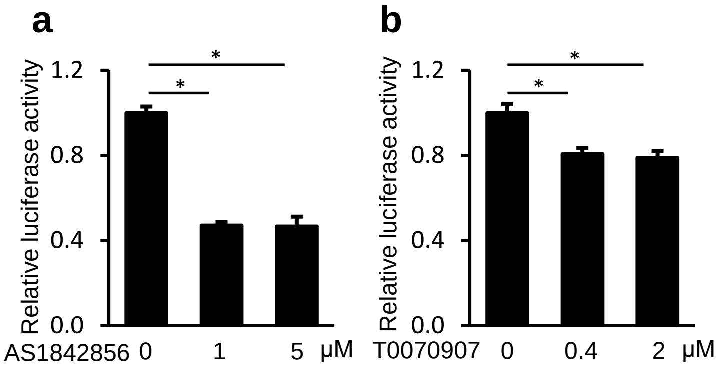

Supplementary Figure S4. ATGL promoter activity is dependent on FoxO1 and PPARγ. HepG2 cells were transfected with -3000/+1-LUC ATGL construct (200 ng) plus pRL-TK-*Renilla* (10 ng). 4 h after transfection, cells were treated with FoxO1 inhibitor, AS1842856 (a) or PPARγ inhibitor, T0070907 (b) with doses as indicated for 60 h before harvesting. ATGL promoter driven luciferase activity were normalized to *Renilla* luciferase activity. Data are expressed as fold-change relative to control and presented as the mean ± SE. *$p < 0.05$.

Supplementary Table S1

Primers used in RT-qPCR for mRNA expression analyses

Gene name	Forward primer	Reverse primer
Pparα	TACTGCCGTTTTCACAAGTGC	AGGTCGTGTTCACAGGTAAGA
Mcad	ACCCTGTGGAGAAGCTGATG	AGCAACAGTGCTTGGAGCTT
Lcad	CACTCAGATATTGTCATGCCCT	TCCATTGAGAATCCAATCACTC
Cpt1α	CTGATGACGGCTATGGTGTTT	GTGAGGCCAAACAAGGTGATA
Cpt2	GCCCAGCTTCCATCTTTACT	CAGGATGTTGTGGTTTATCCGC
Cd36	GAACAGCAGCAAAATCAAGG	AAGACACAGTGTGGTCCTC
Fatp1	CGCTTTCTGCGTATCGTCTG	GATGCACGGGATCGTGTCT
L-fabp	GTGGTCCGCAATGAGTTCAC	GTATTGGTGATTGTGTCTCC
Acsl1	TCTTGGTGTACTACTACGACGAT	CGAGAACCTAAACAAGGACCATT
Acox1	TAACTTCCTCACTCGAAGCCA	AGTTCCATGACCCATCTCTGTC
Acaa1b	CAAGCCCGCGTCCTTAATTC	AGGTGACCCAGCACTACCT
Acaa2	GATCTCAAGCTGGAAGATAC	ACCTCTGCTGAGACTGCAAG
Atgl	TTCACCATCCGCTTGTTGGAG	AGATGGTCACCCAATTTCCTC
Hsl	GCTGGGCTGTCAAGCACTGT	GTAACTGGGTAGGCTGCCAT
Srebp1c	AACGTCACTTCCAGCTAGAC	CCAC-TAAGGTGCCTACAGAGC
Pparγ	GGAAAGACAACGGACAAATCAC	TACGGATCGAAACTGGCAC
Acly	ACCCTTTCACTGGGGATCACA	GACAGGGATCAGGATTTCCTTG
Fasn	TTGACGGCTCACACACCTAC	CGATCTTCCAGGCTCTTCAG
Acc1	ATGGGCGGAATGGTCTCTTTC	TGGGGACCTTGTCTTCATCAT
Acc2	CGCTCACCAACAGTAAGGTGG	GCTTGGCAGGGAGTTCCTC
Scd1	GAAGTCCACGCTCGATCTCA	TGGAGATCTCTTGGAGCATGTG
Mtgpat1	ACGCTGA-GAGTGCCACATACT	GAGAGATCGCTAC-AGCACCAC
Dgat1	CGTGGTATCCTGA-ATTGGTG	GGCGCTTCTCAATCTGAAAT
Dgat2	ATCTTCTCTGTCACCTGGCT	ACCTTTCTTGGGCGTGTTCC
Apob	CCAGAGTGTGGAGCTGAATGT	TTGCTTTTTAGGGAGCCTAGC
Mttp	AGCCAGTGGGCATAGAAAATC	GGTCACTTTACAATCCCCAGAG
Apoc2	ACCTGTACCAGAAGACATACCC	CCTGCGTAAGTGCTCATGG
Apoc3	AGGCTACTGGAGCAAGTTTACT	ATAGCTGGAGTTGGTTGGTCC
Vldlr	AGAGCCTGCCTCCATAGCTG	CGCCCCAGTCTGACCAGTAA
SRA(mouse)	GGCGGGCTGGTGGTACTCG	GCGTCGGCTGATATCATCACATACC
SRA(human)	GCTAGGGCACTAGGTTGTCGC	CGCCTGGCACTGCTGCAGGAAC
36B4	AAGCGCGTCCTGGCATTGTCT	CCGCAGGGGCAGCAGTGG

Supplementary material to Figure 1b

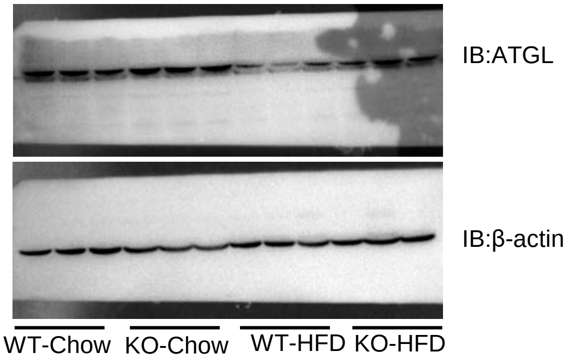

Supplementary material to Figure 2a

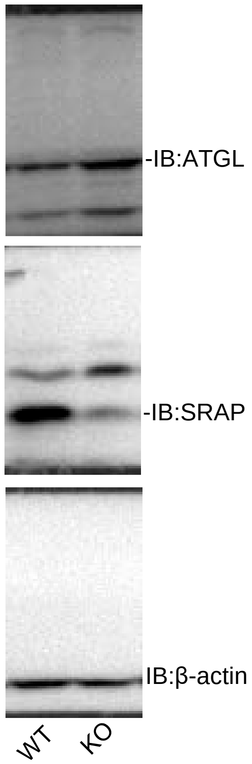

Supplementary material to Figure 2b

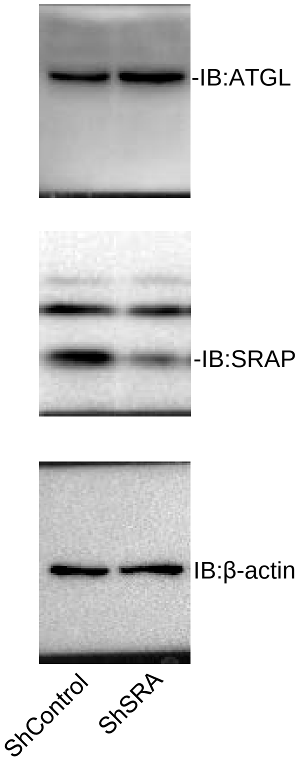

Supplementary material to Figure 2c

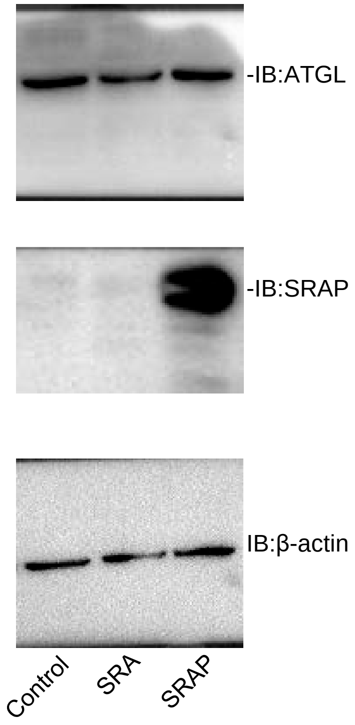

Supplementary material to Figure 3b

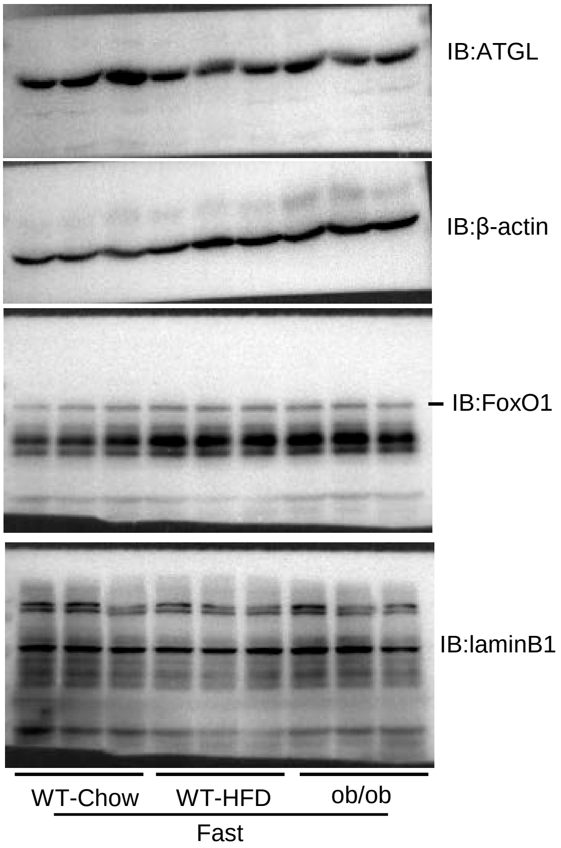

Supplementary material to Figure 3c

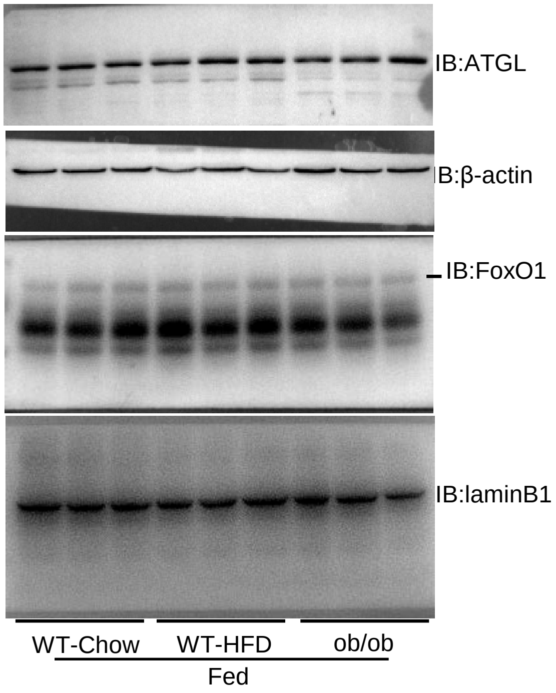

Supplementary material to Figure 3d

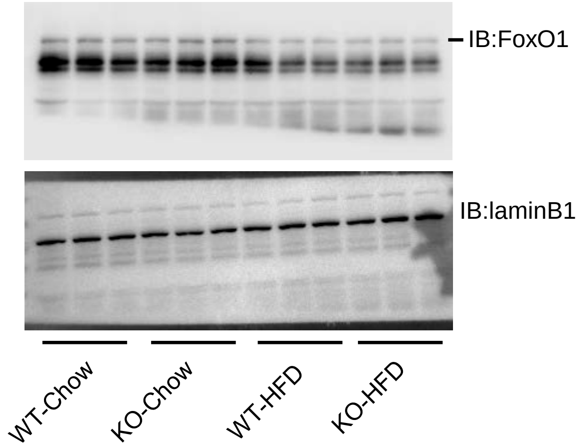

Supplementary material to Figure 4a

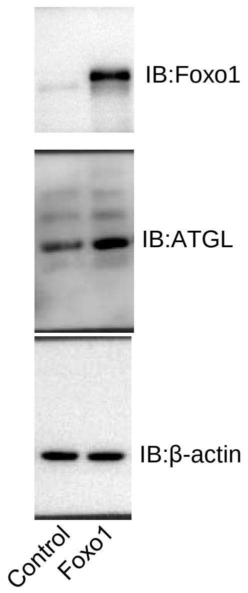

Supplementary material to Figure 5a

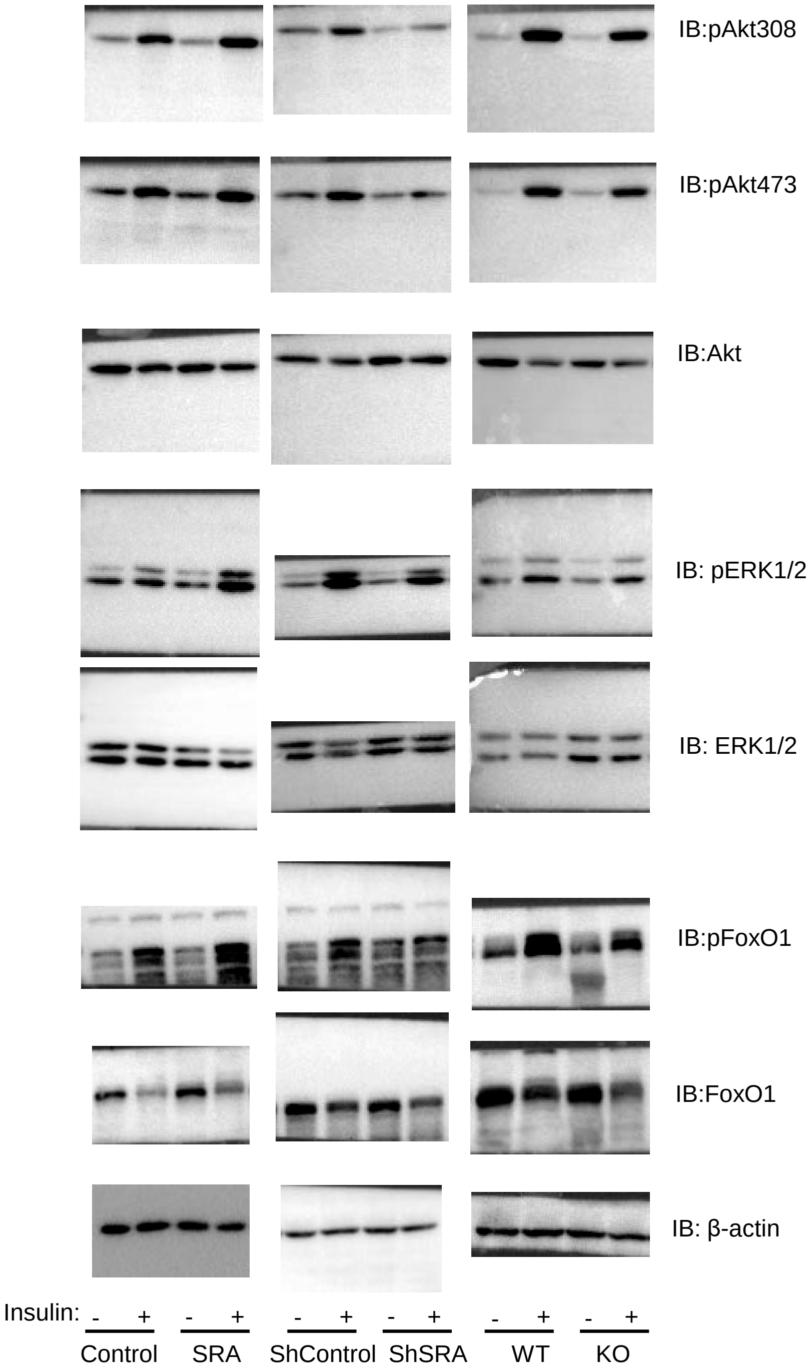

Supplementary material to Figure 5b

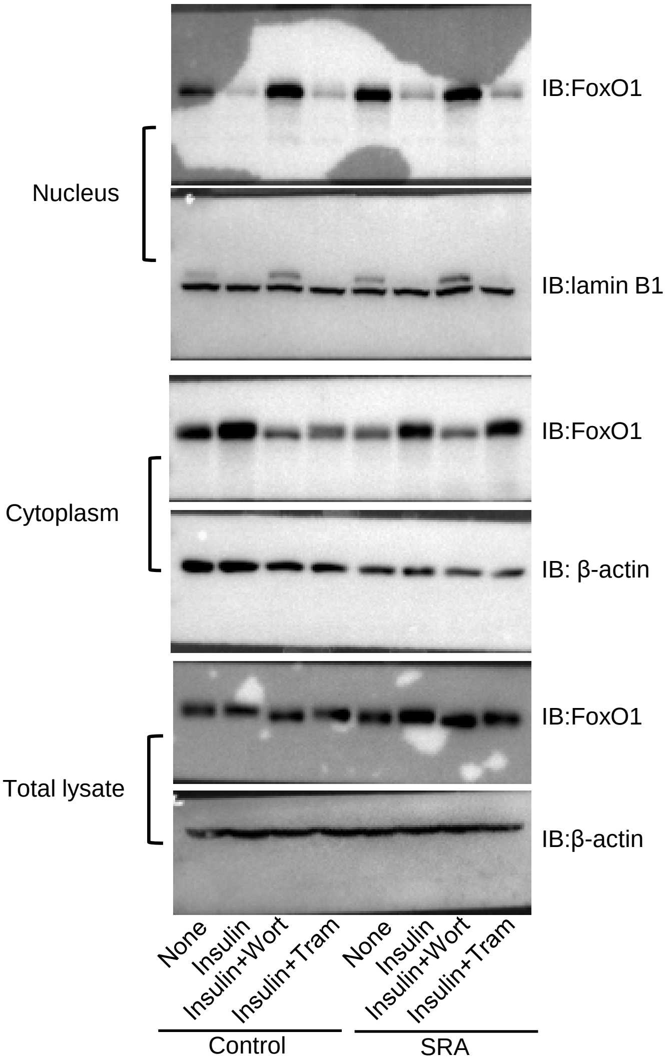

Supplementary material to Figure 7a&b

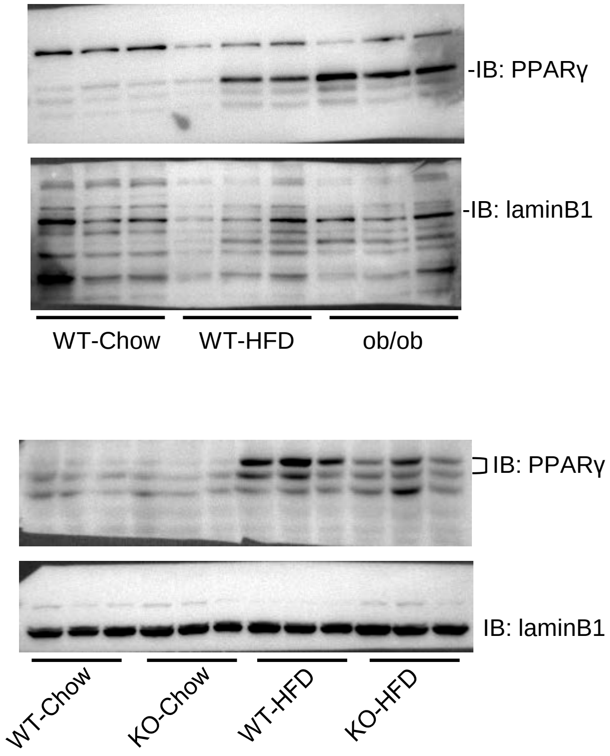

Supplement: Supplementary Information [file srep35531-s1.pdf]
